# State of the Art in Gold Nanoparticle Synthesisation via Pulsed Laser Ablation in Liquid and Its Characterisation for Molecular Imaging: A Review

**DOI:** 10.3390/ma15030875

**Published:** 2022-01-24

**Authors:** Siti Zaleha Mat Isa, Rafidah Zainon, Mahbubunnabi Tamal

**Affiliations:** 1 Department of Biomedical Imaging, Advanced Medical and Dental Institute, Universiti Sains Malaysia, SAINS@BERTAM, Kepala Batas 13200, Pulau Pinang, Malaysia; sitizalehamatisa@student.usm.my; 2 Department of Biomedical Engineering, College of Engineering, Imam Abdulrahman Bin Faisal University, P.O. Box 1982, Dammam 31441, Saudi Arabia; mtamal@iau.edu.sa

**Keywords:** gold nanoparticle, synthesisation, pulsed laser ablation in liquid, characterisation, molecular imaging

## Abstract

With recent advances in nanotechnology, various nanomaterials have been used as drug carriers in molecular imaging for the treatment of cancer. The unique physiochemical properties and biocompatibility of gold nanoparticles have developed a breakthrough in molecular imaging, which allows exploration of gold nanoparticles in drug delivery for diagnostic purpose. The conventional gold nanoparticles synthetisation methods have limitations with chemical contaminations during the synthesisation process and the use of higher energy. Thus, various innovative approaches in gold nanoparticles synthetisation are under development. Recently, studies have been focused on the development of eco-friendly, non-toxic, cost-effective and simple gold nanoparticle synthesisation. The pulsed laser ablation in liquid (PLAL) technique is a versatile synthetic and convincing technique due to its high efficiency, eco-friendly and facile method to produce gold nanoparticle. Therefore, this study aimed to review the eco-friendly gold nanoparticle synthesisation method via the PLAL method and to characterise the gold nanoparticles properties for molecular imaging. This review paper provides new insight to understand the PLAL technique in producing gold nanoparticles and the PLAL parameters that affect gold nanoparticle properties to meet the desired needs in molecular imaging.

## 1. Introduction

Recent advances in nanotechnology and nanomaterials offer many possibilities to explore the nanoparticles as a theranostic function in molecular imaging. Gold nanoparticles have been extensively developed as contrast agent and drug delivery in molecular imaging. This is due to remarkable properties of gold nanoparticles including excellent biocompatibility with human cells, easy bioconjugation with various molecules and chemical stability. Gold nanoparticles can be produced through various methods including conventional chemical synthesis, radiation technologies, electrochemical, biosynthesising and pulsed laser ablation in liquid (PLAL) [1,2].

Despite various methods in gold nanoparticle synthetisation, each method has its own advantages and limitations. Table 1 summarises the gold nanoparticles synthetisation, advantages and limitation. Recently, environmentally friendly method became more popular in gold nanoparticles synthetisation because people are more conscious about various environmental issues. 

Moreover, the PLAL technique is gaining more importance due to its simplicity, rapid rate of formation of gold nanoparticles and eco-friendly approach. Understanding on the PLAL technique is very important to ensure that the gold nanoparticle production meets the desired application in molecular imaging.

PLAL is the most popular technique in nanomaterial formation due to its cost-effectiveness, high efficiency, eco-friendly and facile method [3,4,5]. Alluhaybi et al. also emphasised that the PLAL technique is an excellent method in providing high stability, quality and purity of gold nanoparticles without surface contamination [6]. 

In gold nanoparticle synthesisation, the solid gold target is immersed in the liquid medium. The laser beam ablates the target surface through the liquid medium and deposits energy onto the target that induces local heating and melting of the target material. Ejection and evaporation mechanisms occur at the target materials’ surface and emitted atoms, clusters and droplets then cooldown as gold nanoparticles. The reaction between these two materials produces gold nanoparticles in liquid solution form.

In the PLAL method, there is no reduction of a chemical agent or toxicity surfactants used. Therefore, the PLAL technique maintains the gold nanoparticle purity, quality and homogeneity [7,8,9]. The purity of gold nanoparticles and their larger unique surface (contamination-free) provide a better platform and flexibility for direct conjugating with biocompatible polymers [4,7,10]. 

This purity also resulted in high stability that maintains its shape and chemical properties for up to several years at a room temperature of 26–27 °C synthesised gold nanoparticle [11,12]. The main issues related to PLAL method include sustainability, stability, plasmonic response, reproducibility and portability, interfering by-products, shape control, size control, functionalisation and surface cleanliness.

**Table 1:** A summary of gold nanoparticle synthetisation methods and mechanism, advantages and limitations.

No.	Methods and Mechanism	Advantages	Limitations	Minimum Size of Synthesised Gold Nanoparticle (nm)	Reference
1	Conventional chemical synthesis				
	The chemical reaction involved reduction agents such as citrate and borohydrides in the aqueous medium. These agents reduce gold ions, Au^3+^ (auric) and Au^+^ (aurous), to the non-oxidised stage (Au^0^). This method was adapted from the Turkevich procedure. In addition, stabilising agents such as trisodium citrate dihydrate and polyvinyl alcohol were added to the solution to prevent aggregation of nanoparticle and control the growth.	(i) Simple and easy to synthesise with controllable size and stability of colloidal nanoparticles (ii) It provides a spherical shape of gold nanoparticles with a narrow size distribution. (iii) Able to provide a large number of gold nanoparticles	(i) It is unsuitable for biomedical applications because it uses a toxicity reagent. (ii) It is too sensitive to multiple factors. E.g., an unwashed pipet tip can cause additional foreign material. (iii) Size and surface charge of gold nanoparticles determine absorption across intestinal barriers and accumulation in second target organs after oral administration.	10 nm	[1,2,13,14]
2	Ionising radiation				
	It involves high-energy charge (electron and ion), photon, gamma-ray and X-ray. Gold nanoparticles were synthesised using two different techniques involving direct and indirect effects. Direct effect attributes the energy transfer from radiation, and indirect effect involves the interaction of radical or reactive species generated over the solvent molecule.	(i) It requires proper control of nucleation process by controlling the dose rate. (ii) It does not involve a reducing agent. (iii) It requires high sterilisation and purity and produces narrowed size particles distribution. iv) Faster process and able to produce big amount of gold nanoparticles	(i) Low availability and restrict access to the facilities of gamma irradiator, electron beam accelerator and X-ray device (ii) Difficulty in tagging gold nanoparticle with several materials such as capping or stabilising agents due to sensitivity to high-energy irradiation iii) The particle size depends on a room temperature of 26–27 °C aqueous solution.	7–10 nm	[14,15]
3	Electrochemical				
	Two-electrode cells from gold layer (anode) and platinum layer (cathode) were immersed in the electrolyte solution. The solution contains Hexadecyltrimethylammonium Bromide (CTAB), Tetradodecylammonium Bromide (TCAB) and Tetra Alky Ammonium salts as stabilisers. During electrolysis, by applying electric current, the anode was oxidated to AuBr ions and moved towards cathode. The reduction occurs at cathode. This method synthesises nanorods shape of gold nanoparticles.	(i) Modest equipment (ii) Low cost iii) Lower processing temperature (iv) High quality (v) Ease to control the parameters by adjusting electrodeposition potential, time and concentration of precursor solution	(i) Irreversible self-agglomerations (ii) Less colloidal stability (iii) Poor reliability/repeatability (iv) Non-specificity	1–5 nm	[2,16,17,18]
4	Biosynthesise				
	The biochemical process uses microorganisms, enzymes and plant tissues for biosynthesis for metal nanoparticles. The biochemical processes in biological agents reduce the dissolved metal ions into nano metals. This extract component was mixed with metal salt at a room temperature of 26–27 °C for few minutes to react and stabilised by a non-toxic stabiliser agent. The incubation time is up to a few hours depending on the reaction. The shape of synthesised gold nanoparticle is determined by the concentration of extract, pH, temperature, incubation time and metal salt concentration.	(i) Eco-friendly method (ii) Green approach (iii) Cost-effective	(i) Suitable for medical use (ii) Difficulty in controlling nanoparticle morphology (shape and size), sustainability and not reproducible	5–15 nm	[2,15,16,17]
5	Pulsed laser ablation in liquid (PLAL)				
	PLAL is a versatile synthetic technique that rapidly produces nanoparticles from simple precursor materials by focusing an intense laser beam into a liquid or onto a solid–liquid interface.	(i) Eco-friendly method with minimum operation (ii) Cost-effective (iii) Reproducible process to obtain the desired gold nanoparticle, non-toxicity, high purity and biocompatibility (iv) Minimum waste production during the synthesisation.	(i) Synthesise small amount of gold nanoparticles production. The maximum amount is 4^−10^ g of gold nanoparticles at one time. (ii) Controlling the average size and size distribution	<5 nm	[1]

## 2. The PLAL Mechanism

The PLAL mechanism involves multiple physical processes. During PLAL process, photon energy from the laser is absorbed by the metal target and produced the heating and photoionisation at the irradiated area. The ablation laser energy is converted to the excitation of the electron bonding in the metal target and break the bonding at the threshold energy level. Based on the Inverse Bremsstrahlung principle, these free electrons absorb incoming laser photons and induce further ionisation to the target material [19]. In addition, boiling, vaporisation and explosive process also occurred simultaneously.

Some of the metal surfaces are extracted as vapours, liquid drops, solid fragmentation, or plasma plume. The amount of ablated area is dependent on the absorbed energy, *E.* The estimation of the amount is formulated as shown in Equation (1).

τ_a_ >> τ_1,_
*L*_a_ α *E*^2/3^, τ_a_ α *E*^1/2^, τ_e_ α *E*^1/3^ (1)

where τ_1_ is the laser duration, *L*_a_ is the ablation depth, τ_a_ is the duration of the ablation process and τ_e_ is the electronic temperature during the ablation process. The absorbed energy by the metal target is not constant in time and not uniform on the metal surface [20]. 

The dense and high energetic nonequilibrium plasma are produced from the atomisation and ionisation process at supersonic velocity. Due to its fast expansion, the plasma acts similar to a piston against the surrounding environment, generating shock waves that travel in two opposite directions, in the solid target on one side and through the liquid on the other. This shockwave has induced temperature and pressure increment within the plume and propagates through the target materials [21] The maximum pressure in inward shockwave can be described by the Equation (2).

(2) $$\sqrt{\frac{\alpha }{\alpha +3\;}\times \;\sqrt{z}\;\times \;\sqrt{Io}}$$

where *α* is the interaction efficiency, *z* is the reduced impedance (gcm^−2^s^−1^) and *I_o_* is the laser irradiance (GW cm^−2^)

The high temperature and pressure plasma continuously interact with target materials, and it causes another ablation and enhances the plasma formation [20]. The shockwave induced by nanosecond pulse, may last for several hundreds of microseconds in water up to a few millimetres in diameter before collapsing during cavitation as shown in A study carried out by A. De Giacomo et al. showed that outward shockwave does not have a significant role in the cavitation effect. This is due to non-transition energy from high temperature shockwave outward to the liquid medium [19]. 

During the expansion, the plasma plume cools down and releases energy to the liquid medium. The plasma plume is commonly extinguished after 10^−8^ to 10^−7^ s. This process forms a thin layer of vapour around the plasma volume, generates a cavitation bubble on a time scale of 10^−7^ to 10^−6^ and collapses on a time scale 10^−4^ s [22]. The bubble travels in the liquid up to the maximum diameter in millimetre size. S. Ibrahimkutty et al. emphasised that the cavitation bubble is generated due to interaction of ablation plume within liquid medium [21].

While travelling, the temperature and internal pressure in the bubble decreases less than the liquid surrounding it. Then, the bubble collapses and releases energy by emitting a second shockwave and possibly affect the phase transition and aggregation of nanoparticles produced. After the collapse of the bubble at a time scale 10^−4^, the system reaches a steady in physical and chemical state. However, there is a possibility of minimal modification on nanoparticles produced due to the condensation of ablation atoms and clusters existing on surrounding [22]. There is also a possibility agglomeration occurred for non-stabile particles produced depending on the particle composition and surface oxidation. 

This cavitation bubble acts as a platform for nanoparticle nucleation, growth, coalescence and solidification or more distinct nanoparticles. This bubble plays an important role in confining the primary particles and redeposits to the substrates or nanoparticle [21]. The bubble interaction with the enclosed particles is a critical step in defining primary particles size. The nucleation process occurred following by nuclei growth, coalescence and forming the typical polycrystalline structure. 

There are several parameters that affect the result of nanoparticle synthesis [8,22]. Short laser wavelength increases the absorption energy by metal target and increases the nanoparticles produced. However, the effect is not significant due to the reabsorption effect that increases the efficiency of the ablation, especially in noble metal materials such as silver, gold and platinum. 

This is due to the plasmon resonance properties in UV visible interval, where the new nanoparticle synthesise in the liquid medium is able to reabsorb incoming laser pulse with the double negative effect that decreases the ablation rate and broadens the size distribution. The plasma plume produced is also able to reabsorb the incoming short wavelength laser and introduced to the nonlinearity among ablation and absorption efficiency or laser fluence. However, this reabsorption effect could be eliminated by using near infrared laser wavelength. 

The energy threshold also contributes to the ablation efficiency. Ablation energy thresholds refer to the specific minimum fluences (optical energy per area per pulse) required to remove material from the irradiated area in target metal and generate plasma formation. Higher pulse energy causes larger material to detach from the metal and increase the concentration of the target metal in the plume as well as the energy in the solid and melted fragmentation detachment. This may induce multiple mechanisms simultaneously thermal mechanisms at the edge of the crater and fragmentation at the centre of the spot. Multiple ablation mechanism results in bimodal size distribution produced. Therefore, lower energy is used to produce monomodal size distribution in laser ablation [21]. In addition, higher fluences also expand bubble lifetime and reach maximum radius as shown in Equation (3).

(3) $$Rmax=\frac{tcollapse}{0.915\sqrt{\rho /\left(Pinf-\mathrm{Pv}\right)}}$$

where, *Rmax* is the radius at the maximum bubble, ρ is density fluid, tcollapse is collapse time and Pin and Pv are the liquid pressure and vapour saturation pressure inside the bubble.

In addition, laser pulse duration also plays and an important role in PLAL results. In femtosecond laser pulse (10^−15^), laser energy is released to the electron in the metal target faster than the electron-phonon thermalisation process of the target. Compared to the picosecond (10^−12^) and nanosecond (10^−9^) laser, by thermal relaxation process where the energy is released to the liquid medium before the end of the pulse. In a few tenths of a picosecond, laser irradiation the plasma is generated and lasted for tens of nanoseconds after the ablation. Therefore, no temporal overlapped occurred between ejected material and laser pulse in picosecond and femtosecond laser. However, in nanosecond laser pulse, there is overlap in ablated material and ablation due to the heat conduction.

A longer laser pulse absorbs the incoming laser energy in the plasma plume and increases the plasma temperature and pressure. Then, the plasma atomises the material contained in the plume. This process homogenises the material ejected from the targets [20]. The absorbed energy by the metal target decreases as the plasma plumes produced provided optical shielding around the metal target. However, this phenomenon increases the plasma temperature and enhance the ablation interaction between plasma plume and metal target. For noble material, 10^5^ laser pulses contributed to 1 mg NMPs [20].

In addition, physical-chemical properties of solvent and solutes in the liquid medium also contributes to the nanoparticles produced [23]. Modification of viscosity, density, and surface tension of the solvent in the liquid affects the cavitation bubble and confinement of the plasma plume on the crater. Increasing the viscosity for example by adding ethanol in the liquid medium increases the ablation efficiency by improving the plasma plume confinement on the crater [22] and also increases nanoparticles solution stability [24] by reducing the aggregation [25]. The bio conjugated of nanoparticles can easily achieved with an additional thiol spacer or NH_2_ group during the laser ablation in situ [26].

The temperature and pressure of liquid medium are used to bring the impact to the laser irradiation efficiency, plasma confinement and the cavitation bubble dynamics. This is due to the changes in liquid medium physical including viscosity, density, refractive index, surface tension and compressibility. Increasing pressure increases the cooling plasma rate and decreases cavitation bubble diameter and lifetime. This might cause a defect to the particles synthesised [22]. 

## 3. The PLAL Synthetisation Method

A recent study reported that the most promising and convincing technique in producing gold nanoparticles is by using the PLAL technique [6]. Some studies used pulse laser Neodymium-doped Yttrium Aluminium Garnet (Nd:YAG) to produce gold nanoparticles in the PLAL method [18,27,28,29,30,31,32,33]. The Nd:YAG laser was selected due to its efficiency and wide availability. Green laser with 532 nm wavelength and near-infrared (NIR) laser with 1064 nm wavelength were also used for gold nanoparticles production in the PLAL method. There are several factors that affect the characteristics of gold nanoparticle formation from the PLAL synthetisation method.

### 3.1. Laser Parameters

The laser wavelength, energy, fluence, pulse repetition and time ablation affect the shape, structure, diameter size of synthesised gold nanoparticles and its distribution. The morphology of gold nanoparticles produced by PLAL was in spherical shape. This shape is very important in offering a large surface area for conjugation with other molecules such as peptides, biomarkers and drugs in molecular imaging due to the large surface provided. In addition, the diameter of gold nanoparticle refers to the size of the particles, and the size distribution of gold nanoparticle refers to the amount and pattern of gold nanoparticle in a liquid medium such as uniform or clusters.

There are two common types of laser ablation wavelengths used in the previous studies to synthesise gold nanoparticles using the PLAL technique. It includes NIR laser with 1064 nm and green laser with 532 or 355 nm wavelength. Alluhaybi et al. [6] reported that laser wavelength plays an important role in controlling the shape, size, stability and growth of gold nanoparticles [12]. The absorption energy by metal target is increased with lower laser ablation wavelength. The interband transition in metal targets and strong plasmon (for gold material) from nanocomposites with other particles in the same liquid medium also increases the absorption energy by target material. These will increase large fragmentation from metal targets and produce smaller size distribution with smaller average diameter sizes of the particles [22,32].

Other than that, the ablation laser at 555 nm wavelength also produced more homogenous erosion on the target surface than the rugged erosion at 1064 nm wavelength at the same fluences. The crater morphology of metal target correlated to the homogeneity product produced which is bring implication to the particles size, size distribution, surface, and inner composition of nanoparticles. The homogenous erosion produced smaller average diameters (3–6 nm) due to the vaporisation process, and the opposite character produced larger size of nanoparticles with size distribution peaks at 10 nm due to the explosive boiling process of the target surface [20].

**Figure 1:**
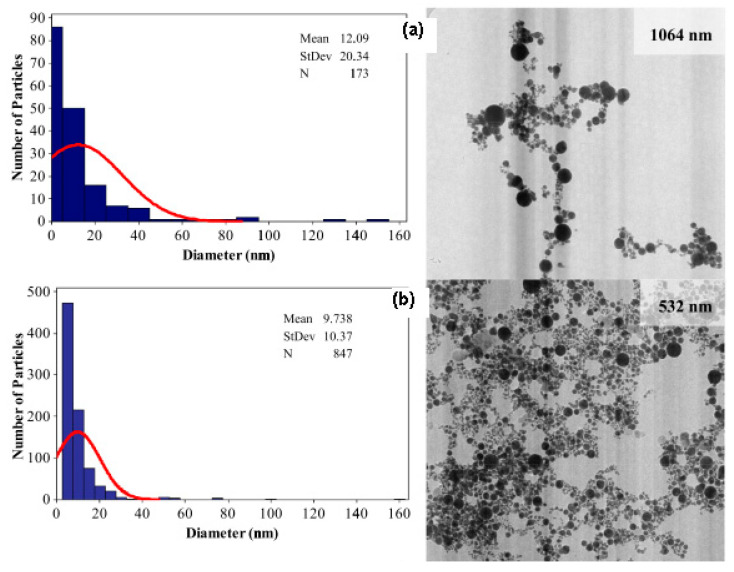
Histogram of the size distribution on TEM images of the gold nanoparticles produced by (**a**) 1064 nm and (**b**) 532 nm in distilled water (retrieved with permission from reference [33]).

Figure 1 shows that short laser wavelength (532 nm) has higher photon energy and it is able to produce smaller nanoparticle diameter with mean diameter size below 10 nm and smaller size distribution. Therefore, the selection of appropriate laser wavelength with minimum absorption depth is very important to ensure the efficient and quick ablation outcome with high-energy deposition within a small volume [8].

Furthermore, laser energy contributes to the size of the resultant gold nanoparticle and amount of ablated material. The laser energy is measured in mJ. The linear decay of the ablation rate with spot diameter leads to the inverse-proportional increase of the ablation rate with the square root of the laser fluence [6,20]. An increment in laser energy enhances the number of synthesised gold nanoparticles with larger size distribution, larger nanoparticle sizes and higher solution concentration [33]. However, this effects for materials with lower reflectivity [33]. Enhancing the power ablation to the gold target material will cause a higher detachment of target material, formation of plume and nanoparticles dissolved in a liquid medium and increase solution concentration. High concentration of gold nanoparticles by the PLAL method visually shows a darker colour due to surface plasmon resonance (SPR) characteristic. 

Apart from fragmentation, explosive boiling and vaporisation that occur simultaneously, there is high probability occurrence of thermal mechanism at the edge of the crater and fragmentation at the centre part of the laser spot. Due to the multiple ablation mechanism, bimodal size distribution is obtained in high laser energy compared with monomodal in lower energy prospectively.

**Figure 2:**
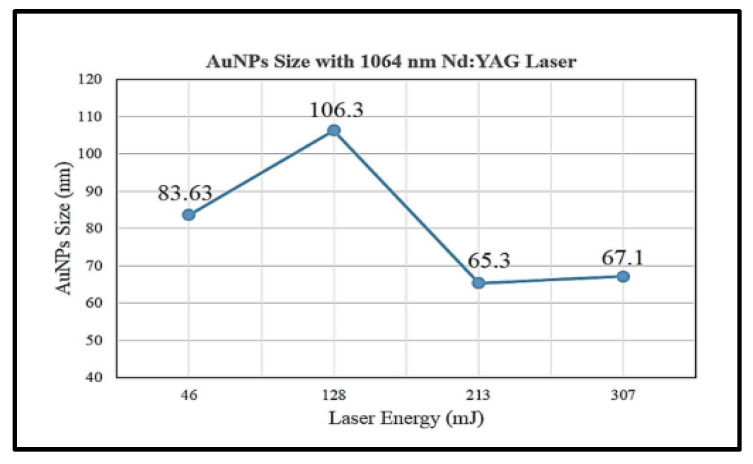
Average size of gold nanoparticles synthesised by 1064 nm wavelength laser ablation at energy of 45, 128, 213 and 307 mJ (retrieved with permission from reference [32]).

A study shows that higher laser energy at 318 mJ will produce smaller average particle sizes of gold nanoparticle (7.516 nm) than that at 286, 226 and 96.6 mJ with particle sizes at 17.5, 25 and 30 nm, respectively, with the same 1064 nm laser ablation wavelength [6]. However, this is in disagreement with a study performed by Jamaludin et al. [32] as shown in Figure 2.

Figure 2 shows that the average size of gold nanoparticles synthesised was decreased with increasing laser ablation energy at 128 and 231 mJ with particle sizes of 106.3 and 65.3 nm, respectively. However, the particle size with a laser energy of 46 mJ is smaller than that of 128 mJ with 83.63 and 106.3 nm, respectively. Hernandez-Maya et al. emphasised that the optimal energy used for laser ablation ranges between 100 and 180 mJ. The authors also claimed that it was not possible to ablate the target material with energy less than 100 mJ and difficult to reproduce gold nanoparticles with energy more than 180 mJ [5]. The study also reported that saturation tendency might occur in gold nanoparticle solution and a possibility of gold debris present in the solution [5].

Another laser parameter that affects the characteristics of synthesised gold nanoparticles in the PLAL technique is laser fluences. Laser fluence refers to the time-integrated flux laser to the target. It corresponds to laser energy and is measured in J cm^2^. The higher the laser energy, the greater the laser fluences produced with the same size of the targeted area involved. Therefore, higher laser fluences will produce a smaller diameter size of gold nanoparticle produced. Higher fluences ablation laser for green laser and NIR laser wavelengths will produce a rugged crater on the metal target surface. Thus, 60 J cm^−1^ produced sharper craters, whereas 1000 J cm^−1^ produced irregular craters. Homogenous craters surface and low fluences of laser ablation produced will produced small size nanoparticles diameters and distribution [20].

Furthermore, laser pulse repetition rate showed a significant effect on the development of gold nanoparticles. Repetition rate refers to the number of laser pulse per second that hits the target. Higher pulse repetition will produce more gold nanoparticles. This linear correlation occurred in the range of 10^−4^–10^4^ s or 10^3^–10^4^ Hz, where the repetition rate is longer than the lifetime bubble cavitation time [22]. The bubble cavitation reduces the laser ablation to the metal target and causes scattering of laser light. During the expansion of bubble cavitation, laser ablation occurs in the low density hot gaseous phase. Therefore, confined plasma plume on the crater surface is less efficient on the detached metal target.

A high pulse repetition rate will maintain the heat produced by ablation and reduce energy waste. This situation speeds up aggregation and coalescence and increases the amount and concentration of synthesised gold nanoparticles [6]. A study shows that increasing the pulse repetition rate at 8 Hz reduced the average size of particle size to 3.29 nm compared with 17.54 nm at 2 Hz [6] Other than that, at higher repetition rate (kHz), the metal target will increase higher (26–27 °C) than the room temperature and reaches the energy threshold for detachment metal surface [22].

Other than that, the ablation duration in the PLAL method also plays a role in nanoparticles synthesised. A longer ablation duration will increase the amount of ablated metal target due to the increase of temperature, and the pressure generated in plasma plume. Continues ablation will produces a smaller size of gold nanoparticles and increasing the concentration of gold particles in a liquid medium [7]. Table 2 shows that 10 min ablation produced gold nanoparticle with a diameter size of 24–9 nm and 5 min ablation produced 21–14 nm gold nanoparticle with energy range 120 mJ–140 mJ [5].

However, shorter pulse duration minimised the thermal damage that is inflicted on the surroundings, and it has high possibility of performing ablation at maximum peak power.

### 3.2. Liquid Medium

There are various types of liquid medium used to immerse the target metal (solid gold) during the PLAL method. The selection of the liquid medium depends on the type of gold nanoparticle production to produce either hybrid (gold coating with other metals such as iron oxide nanoparticles) or pure gold nanoparticle [34].

Deionised or pure water was used to produce pure gold nanoparticles [6,35,36]. The ionic solvent was used as a capping agent, template and precursor in gold nanoparticle synthetisation [4,37,38]. Compared with other metals, the gold nanoparticles can be synthesised stable either in water or organic solvents without stabilising molecules or ligand using the PLAL method [23]. The stability and nanoparticle size in liquid could be controlled by adjusting the ionic strength without compromising the nanoparticle adsorption of the supporting material.

A study shows that palladium nanoparticles in pure water have weak stability where the particles were precipitated with mass loss approximately 30% after 50 min of ablation times [23]. Extra salt in saline acts as an oxidising agent that could increase the stability by reducing the aggregation effect and preventing particle growth. Therefore, the particle sizes produced are smaller. In addition, the stability of nanoparticles could be increased by ablated material in highly diluted electrolytes medium [23]. Meanwhile, the high stability of nanoparticles in deionised water is reported related to the surface charge of the particles causing a zeta potential and electrostatic repulsion of the particles [39].

In certain condition, the stabilisers such as thiols, citrate and other ligands were added to the gold particle during the ablation process. This stabiliser will dump on gold nanoparticles surface and caused a negative effect to the gold nanoparticle plasmonic properties. It will decrease the nanoparticle sizes by inhibit agglomeration process [22,23].

Another study reported that liquid media volume used to immerse the target metal does not affect the ablation duration and physiochemical property of gold nanoparticle with respect to morphology, structure and its function [5,7]. These findings were proven in the fabrication of gold nanoparticles and other nanoalloys such as argentum (Ag), stannic oxide and silicon [3,40,41,42] via the PLAL technique. In addition, with the use of a small amount of liquid media and gold target, the number of gold nanoparticles obtained did not exceed 10^−4^ g [5].

Table 3 shows that the % mass of gold particles produced reduced with increasing depth liquid medium from 10 to 15 mL from the target surface. Deeper liquid medium will decrease the diameter size of gold nanoparticles, produce uniformity of nanoparticles and narrow the gold nanoparticles distribution [6,37].

On the other hand, a recent study also reported that stirring the liquid medium during the ablation time or immediately a few minutes after the ablation process produced homogeneous gold nanoparticles without any agglomeration in gold nanoparticle solution [37]. There are a few techniques used in stirring by either rotation motor magnetically with constant speed or manual stirring by using a spatula.

Table 4 shows that the stirred liquid medium during the ablation process for 30 min has reduced the diameter size of gold nanoparticles. In addition, the stirring process avoids a crater on the target gold surface and produces a uniform particles size distribution [3,9].

High concentration of nanosolution or ionic solvent decreases laser energy to target metal exponentially with radiation time. The effect of laser parameters and liquid medium in gold nanoparticle synthesis via the PLAL method is summarised in Table 5.

## 4. Gold Nanoparticles in Molecular Imaging

Gold nanoparticles have facilitated breakthroughs in molecular imaging that allow drug delivery and imaging of physiological processes. Molecular imaging is a quantitative, non-invasive imaging method of targeted biomolecules and monitoring of related biological processes in living subjects. It has attracted much attention in disease studies based on molecular marker. Every cell type has its unique molecular signature and identifiable characteristics. Therefore, biomarkers can facilitate the molecular definition of cancer.

Conjugation between gold nanoparticles and targeting biomolecule radionuclide can be tailored ideally to meet the needs in development of the precision and personalised medicine to the specific cancer cells. This conjugating will enhance the interaction with cancer cells surface and increase the sensitivity detection by using molecular imaging modalities. Nanoparticles surface with targeting biomolecules provides specific targeting tumour through interactions with biomarkers in the specific tumour cells or normal cells surrounding.

On the other hand, the nanoparticles also enhanced the permeability and retention (EPR) effect in vascularisation. In a normal body circulatory system, less than 10 nm diameter of nanoparticles can be eliminated from the body through the kidney. However, in cancerous organs, the blood vessels surrounding are immature, and it allows the larger size nanoparticles to pass through the blood vessel membrane cells into the interstitial space of the tumour. Conjugating nanoparticles with non-permeable drug provides an opportunity for delivery of the drug to the cancerous organs and provides better treatment [1]. In this case, the nanoparticles will be used as drug delivery agent.

However, the nanoparticles with a size of more than 100 nm are unable to pass through the vessel membrane. These nanoparticles with a size of more than 100 nm will inhibit the nanoparticle functions as a diagnostic tool and therapy in delivering drug treatment to the targeted cancer cells. In addition, Jahangirian et al. emphasised that nanoparticles within the range of 50–100 nm with a slightly negative charge can penetrate the membrane of cancer via systemic systems [46]. The study showed that 20 nm gold nanoparticles have been conjugated to different cellular targeting peptides and are able to penetrate biological membranes to the nucleus of targeted cancer [1,47].

The nanoparticles can be synthesised from various materials including silver (Ag), iron oxide, zinc oxide, carbon, cadmium selenide and indium phosphide. However, gold nanoparticle has gained high prominence in the biomedicine field due to its unique and extraordinary physicochemical properties that offer different imaging and therapeutic uses as shown in Table 6.

The advances in nanotechnology and combination in high sensitivity of molecular imaging modalities such as positron emission tomography/magnetic resonance imaging (PET/MRI), positron emission tomography/computed tomography (PET/CT) and single photon emission tomography/computed tomography (SPECT/CT) provide better detection and staging for therapeutics.

With the use of imaging technology, the size and concentration of the gold nanoparticles play important roles on the image enhancement and image quality [51]. The main functions of nanoparticle in molecular imaging are as drug delivery agent and therapeutic enhancement. Recent applications of gold nanoparticles in molecular imaging are shown in Table 7.

Therefore, based on the EPR effect and vascularity characterisation, the major concerns in the production of gold nanoparticles for molecular imaging are diameters size, shape, purity, non-toxicity, flexibility in direct conjugating with other chemicals and polymers and the stability at certain period. All of these aspects are crucial in the development of diagnostic and therapeutic tools responsible for their bioavailability and bioaccumulation in a biological system.

## 5. Characterisation of Gold Nanoparticle

The properties of gold nanoparticles synthesised from the PLAL technique depend on the morphological and geometric characteristics of the particles [56,57]. Characterisation of synthesised gold nanoparticles is important to ensure that the gold nanoparticle properties suit the applications such as for sensing, imaging and biomedical therapeutic [15]. The important properties for the characterisation of gold nanoparticles include its physicochemical properties such as the size, shape, specific surface area, agglomeration, aggregation state, size of distribution, surface morphology, the structure including crystallinity and defect structure. In photothermal therapy, spherical and nanorod solid gold nanoparticles with a diameter size larger than 50 nm were preferable due to strong NIR absorption [12,15].

It is reported that the size distribution characterisation is the most challenging in colloidal chemistry and nanotechnology [58]. This is due to the small structure of gold nanoparticles, close to each other or other particles (neighbour) commonly seen as an overlapping image. With the technology growing, there are several analytical and imaging techniques that can be used to fully characterise the synthesised gold nanoparticle. Previous studies have shown that X-ray diffraction (XRD), energy dispersive X-ray analysis (EDX) and ultraviolet (UV)–visible spectroscopy were among the most extensive techniques used to characterise the gold nanoparticle [7,32,35].

### 5.1. X-ray Diffraction

The XRD is one of the most extensively used techniques to characterise the gold nanoparticle. The XRD gives information on the crystalline structure, spatial arrangement of an atom and crystalline grain size. The X-ray was irradiated on the gold nanoparticle, and the intensity of scattering angle of X-ray from gold nanoparticle was measured. According to XRD card (JCPDS no. 65-2870), the remarkable peak for gold nanoparticle was at d at 20 = 38.31°, 44.47°, 64.58° and 77.43°, which is indexed to the planes of (111), (200), (220), and (311) [40,44]. In mixed solution, increasing gold nanoparticle concentration will decrease the intensity peak in the XRD pattern [41].

The XRD was commonly performed in samples of powder form after drying their corresponding colloidal solutions. The advantage of using XRD techniques was it results in statistically representative, volume-averaged values. However, the XRD technique is not suitable for amorphous materials, and the XRD peaks are too broad for particles with a size below 3 nm [59].

### 5.2. Nanoscopic Imaging

Nanoscopic imaging such as the EDX was used in identifying the morphology, elemental composition, concentration and segregation element in the synthesised gold nanoparticle [36,40,59]. This system was attached with scanning electron microscopy (SEM) or transmission electron microscopy (TEM).

In principle, the primary electron beam from EDX hits the inner shell of the gold nanoparticle atom. The high-energy electron from the outer shell fills the inner shell and releases energy in the form of an X-ray. This specific and unique element on each atom molecule was collected by silicon drift detector, and then micrographs of the gold nanoparticle were analysed using the ImageJ tool software program [9].

The data obtained from this test consist of spectra peaks corresponding to the elements in a sample. Therefore, a higher peak in the spectrum shows a higher concentration of synthesised gold nanoparticles. In addition, any element (contamination) in the gold nanoparticle surface can be identified. Micrographs of TEM also provide morphology information such as spherical shape and diameter size of synthesised gold nanoparticles [9,41,59,60]. TEM showed high spectra and spatial resolution of image quality compared to SEM in differentiated agglomeration effect [61].

On the other hand, the EDX technique provides qualitative, semi-qualitative and quantitative data as well as special distribution through element mapping of synthesised gold nanoparticles. However, the EDX is not a precise tool in chemical analysis. It only provides an estimation of the distribution of elements in the solution. Therefore, a combination of XRD and EDS is the best for characterisation of gold nanoparticle. Apart from microscopy and EDX techniques, Fourier-transform infrared (FTIR) microscopy, Raman microscopy, nuclear magnetic resonance spectroscopy, X-ray photoelectron spectroscopy (XPS) and secondary-ion mass spectrometry analyses can also be used in characterising gold nanoparticles.

### 5.3. Atomic Force Microscopy

Atomic force microscopy (AFM) is the most versatile technique in nanoscale studies. It has the ability to provide 3D topography in various types of surface measurements, and it represents extraordinary tools for detailed characterisation of sub-micron size as the surface functionalisation. Other than that, the AFM also generates images at atomic resolution with minimum sample preparation.

### 5.4. UV–Visible Spectroscopy

UV–visible spectroscopy was used to determine the concentration or weight synthesised gold nanoparticle by measuring the UV light absorbed. This is the effective method for real-time monitoring of the surface during the addition of ligands. When gold nanoparticle molecules absorbed energy from UV or visible light, the electron bonding in gold nanoparticles was excited to a higher energy orbit. The gold nanoparticle is an electric conductor; thus, it also produced SPR. Therefore, the absorption intensity in spectrometer will increase with increasing of gold nanoparticles due to the influences of surface plasmon resonance, depending to their size and shape [62]. Different sizes of gold nanoparticles have different specific absorption spectra and result in differences in visible colour [63]. This is in line with previous studies performed. S. H. Lee et al. claimed that the maximum absorption of light for pure gold nanoparticles is 530 nm in the NIR region [35]. This was supported by a study conducted by Kuriakose et al. [7], and Nasiri et al. [41] also found that the maximum absorption light is 522 and 526 nm, respectively. A study performed by Prakash et al. found that the size and shape of synthesised gold nanoparticles affect the surface resonance plasmon characteristic [62]. 

**Figure 3:**
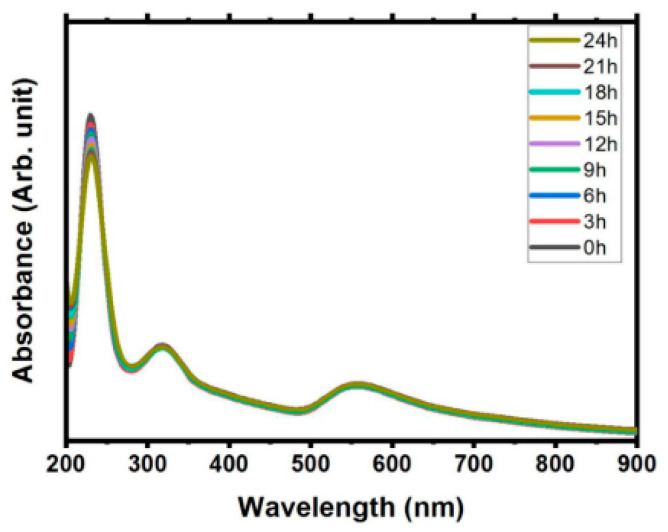
Absorption study from the ablation metal (Gold and Sn) immersed in pure water in time dependent over a day (retrieved with permission from reference [40]).

In addition, the SPR absorbance value does not bring significant changes over the time in stabile solution. Figure 3 shows small shifted in wavelength, with decrease the value of absorbance amplitude recorded as time passed for 24 h [40]. This study also has shown minimal decreased in Zeta potential value, that indicates the good stability solution. Moreover, the SPR determines the visible colour based on the concentration and size of synthesised gold nanoparticles [9].]. The SPR wavelength increases with the particle size and shape and corresponds to the colour of the gold nanoparticle produced. The gold nanoparticle size in a range of 10–20 nm showed red colour, and it changed to blue and purple with the increment of their size [41].

In addition, Naharudin et al. [9] showed that the increment of pulse laser ablation time increases gold nanoparticle concentration and changes the solution colour from light pink to darker. The colourless ultrapure water also changed to ruby red and violet colour with the presence of gold nanoparticles. A summary of characterisation of gold nanoparticles is presented in Table 8.

## 6. Conclusions

In conclusion, the PLAL technique is a green synthetic and promising technique to produce nanoparticles with no toxic reducing agents or organic stabilisers. The PLAL technique produces useful quantities of gold nanoparticles with few minutes of laser irradiation and very little post-synthetic tasks as compared to hours or days of traditional methods. The PLAL method can produce the desired size of gold nanoparticles and spherical shape of the gold nanoparticle that can be achieved by adjusting or selecting suitable laser parameters such as laser wavelength, energy, and repetition rate as well as liquid media. In addition, the EDX, XRD, AFM and UV–visible spectroscopy are widely available and can be used to characterise the synthesised gold nanoparticle via the PLAL method. Gold nanoparticles have revolutionised the nanomedicine field by playing an important role in cancer diagnostics and treatment.

## Figures and Tables

**Table 2:** Particle diameter obtained with 15 mL of water at different time ablation (retrieved with permission from reference [5]).

Time = 5 min		Time = 10 min	
Energy (mJ)	PD (nm)	Energy (mJ)	PD (nm)
120	21	120	9
130	19	130	19
140	14	140	25

**Table 3:** Mass percentage of gold in the sample medium with 10 and 15 mL (retrieved with permission from reference [5]).

	% Mass of Gold	
	t = 5 Min	
Energy	V = 10 mL	V =15 mL
120	4.80	2.88
130	16.75	14.22
140	5.66	22.50

**Table 4:** Comparison of mean particles diameter for stirring and stationary tetrahydrofuran medium for four different times (retrieved with permission from reference [9]).

Condition	Stirred		Stationary	
PLAL Time (min)	Au-Np Size (nm)	% of NPs Population Size >15 nm	Au-Np Size (nm)	% of NPs Population Size >15 nm
7	11.5 (6.9)	28.26	11.5 (5.6)	26.82
10	7.9 (6.6)	13.8	11.0 (6.8)	27.58
15	7.8 (6.6)	10.89	14.0 (10.8)	39.21
30	6.0 (2.6)	0.47	10.07 (7.3)	20.00

**Table 5:** A summary of the effect of laser parameters selection on diameter of gold nanoparticles.

Reference	Laser Wavelength (nm)	Laser Energy (mJ)	Laser Fluence (J/cm^−1^)	Repetition Rate (Hz)	Laser Pulse/Time Ablation	Liquid Medium/Depth (mL)	Average Diameter (nm)	Scaling Instruments
[43]	1064	–	23.96	1	500 pulses	Deionised water/5 mL	7–10	TEM
[11]	532		30	10	30 min	Distilled water/30 mL	13	SEM, TEM
[9]	532	318	–	40	30 min with stirring	THF/20 mL	6	HRTEM
[7]	1064	–	–	10	30 min (stop every 3 min)	Deionised water/10 mL	7.4	TEM, DLS, zeta potential
[5]	532	120	–	10	5 min	Milli-Q water/15 mL	21	SEM, XRD, XPS
					10 min		9	
					5 min	15 mL	2.88	
						10 mL	4.80	
[44]	1064	950	–	5	1000	Distilled water/3 mL	6.09	TEM, X-ray diffraction
						Ethanol/3 mL	24.71	
[41]	1064	1.5 ns	–	10	5000	Deionised water	60	XRD, TEM
[33]	532	950	–	5	1000	Distilled water/3 mL	9.738	TEM
	1064						12.09	
[45]	1064	2.5 ns	–	–	20 min	Deionised water/6 mL	<20	TEM, HRTEM, EDX

**Table 6:** The gold nanoparticle properties and the benefits of molecular imaging.

Reference	Gold Nanoparticle Properties	Benefits of Molecular Imaging
[48]	Versatile structural modification	(i) Easily linked to different chemical components and organic molecules for different functionality and personalisation as targeted delivery (ii) It provides high labelling capacity.
[49]	Biocompatibility and non-toxicity to the human body	(i) An excellent candidate for drug carriers (ii) The nanosised carriers offer an apt means of transporting small molecules and biomacromolecules to diseased cells/tissues. (iii) Resistant to the high temperature, photoirradiation, acids or oxidation
[50]	High atomic number	It has higher potential in providing good contrast agent especially for soft tissues.
[2]	Optical properties due to unique Surface plasmon resonance (SPR)	It has intense absorption and scattering bands in NIR interval. Intense absorption will increase photothermal effect for destructive tissues and cancer cells. Meanwhile, scattering features will increase effectiveness in sensitivity in diagnostic imaging
[47]	Large surface volume ratio	It provides multivalency conjugation for multi-functionality and flexibility components.
[1]	Surface charge	(i) It provides physicochemical stability and further implementation in the cellular process and bioaccumulation. (ii) Positive charge causes cell death at lower concentration, and neutrally charge causes cellular death at significant higher concentration.

**Table 7:** Recent applications of gold nanoparticles in molecular imaging.

Reference	Nanoparticles Complex	Molecular Imaging Modality	Outcomes	Tumour Model/Cell Line
[51]	^99m^Tc-DOTA-Fe_3_O_4_@ Au radiolabelled and Fe_3_O_4_@ Au nanoparticles	MRI, CT and SPECT	Potential multimodal SPECT/CT/MRI imaging contrast agent for imaging gold nanoparticles with a mean diameter of 27 nm, and it is composed of 8 nm iron oxide core and a 9.5 nm thick gold shell.	None
[52]	^99m^Tc-gallic-gold nanoparticles	SPECT	There was an increase uptake of ^99m^Tc-gallic-gold nanoparticles in tumour cells. There was good stability and cytocompatibility in tumour site.	Ehrlich ascites carcinoma in xerograph albino mice
[53]	^99m^Tc-HYNIC-(Tricine/EDDA)-Lys-FROP	Dual head gamma camera	Selective delivery nanoparticles successfully delivered to the specific tumour and improved diagnostic efficiency.	Breast cancer xerograph nude female mouse (MCF-7)
[54]	^111^I-HAuNS (hallow gold nanoparticles)	SPECT/CT	Images showed higher intensity image in the targeted region even after 24 h.	Nude mice xerograph tongues tumour (OSC-19)
[55]	^64^Cu-PEG-HAuNS	PET/CT	High accumulative contrast in the tumour area after 1 h of injection. It is useful for targeted chemotherapy and photoablative therapy.	VX2 liver cancer-bearing rabbits

**Table 8:** A summary of characterisation method of gold nanoparticle, function, advantages and disadvantages of each method.

Characterisation Method	Function	Advantage	Disadvantage
XRD	It determines crystalline structure, spatial arrangement of atom (composition) and crystalline grain size.	It provides a statistical result as representative volume-averaged values.	It is unsuitable for amorphous materials. XRD peaks are too broad for particles with a size below 3 nm.
Nanoscopic imaging	Identifying the morphology, elemental composition, concentration and segregation element in the synthesised gold nanoparticle It detects and localises a nanoparticles diameter size, size monodispersity, shape, aggregation state. It quantifies nanoparticles in matrices and kinetic study. It provides information regarding the crystal structure of single particles by distinguishing monocrystalline, polycrystalline and amorphous particles (for HRTEM only).	It provides morphology information such as shape and diameter size. It provides qualitative, semi-qualitative and quantitative data as well as a special distribution. It is able to detect defects of the nanoparticle structure.	It is not a precise tool in chemical analysis. It provides an estimation data for the distribution of elements in the solution.
Atomic force microscopy (AFM)	It generates an accurate topographic map of the surface features. It measures and localises different forces including adhesion strength, magnetic forces and mechanical properties.	It can be performed in various environments including ambient, gas and liquid. It provides higher resolution in 3D topography at atomic scale. It requires minimum preparation.	It has limited scanning size.
UV–Vis spectrometer	It determines concentration or weight synthesised gold nanoparticle by measuring the UV light absorbed.	It is easy to perform. It provides qualitative data of absorbance peak.	It is applicable in liquid samples It has low sensitivity and is difficult to analyse the liquid concentration.

## Data Availability

The data presented in this study are openly available in [ScienceDirect, IOPScience, ScienceDirect, ProQuest, OpticaPublishing] at [doi:10.1016/j.rinp.2019.102497], [doi:10.1088/1742-6596/1484/1/012029], [doi:10.1016/j.molstruc.2020.128913], [doi:10.1088/1742-6596/1386/1/012062], [doi:10.1364/OME.381427] reference numbers [33], [32], [40], [5], [9], Figure 1, Figure 2, Figure 3, Table 2, Table 3 and Table 4, respectively.
